# Can cancer go green? It’s up to us

**DOI:** 10.3389/fonc.2023.1074091

**Published:** 2023-02-22

**Authors:** Richard J. Epstein, Yanfei Gu, Frank P. Y. Lin

**Affiliations:** ^1^ New Hope Cancer Center, Beijing United Hospital, Beijing, China; ^2^ Department of Medicine, University of New South Wales, Sydney, NSW, Australia; ^3^ Cancer Programme, Garvan Institute of Medical Research, Sydney, NSW, Australia; ^4^ National Health & Medical Research Council (NHMRC) Clinical Trials Centre, Sydney University, Sydney, NSW, Australia

**Keywords:** oncology, health economics, sustainability, demographics, population aging

## Introduction

The problem of cancer has long been supported by public taxation, private philanthropy and business investment (1), with this support having been both a cause and effect of clinical and scientific breakthroughs – including but not limited to adjuvant therapies, targeted therapies, immunotherapies, digitised imaging technologies, genetic sequencing, and cancer-preventive vaccines. Indeed, the term “oncology” only entered professional usage after proclamation of a War on Cancer by President Nixon in the lead-up to his 1972 re-election (2); this anti-cancer campaign was revived in 2016 by the Cancer Moonshot project, “re-ignition” of which was declared in 2022 by President Biden, with the goal of reducing age-specific cancer deaths by 50% over 25 years (3).

To understand the success [notwithstanding certain caveats (4)] of what has so far been a half-century campaign, it should first be asked why cancer has attracted more funding per unit of disease-specific mortality than have most other health issues; for example, there has been no similar support for a War on Heart Disease, even though cardiovascular problems have long caused higher death rates and health costs than cancer (5). This depth of support for cancer has been attributed to perceptions that a cancer diagnosis presents a unique existential threat [i.e., an “unspeakable” illness (6)] that not only poses lethal risks but also creates spiritually arduous – whether “moralistic” or “militaristic” – uncertainties as to the timing of cancer recurrence, nature of future symptoms, disease response or resistance, and speed (rapidity or slowness) of death (7).

Nonetheless, since resources are limited in any system, even the most serious personal health concerns (8, 9) must ultimately compete for support with societal-level threats (10, 11). During the first two decades (1971-90) of the War on Cancer, such threats included overpopulation, risks of nuclear war, and the HIV pandemic. Concerns over these issues abated over time; in hindsight, this fading of competing threats – akin to a peace dividend (12) – enabled sharper focus on the individual risk of a cancer diagnosis. Looking ahead, although cancer will always loom large as a major worry for personal health (13), the association of this disease with aging [i.e., with a low detriment to species fertility (14)] ensures that its impact on humanity will remain modest (15).

## End of the golden age

Competing threats to the traditional support base for cancer lie ahead. Amongst these are the approaching impacts of global population aging, in tandem with falling birth rates (16), on oncology practice. Population aging will increase the aggregate burden of cancer diagnoses (17), even as age-specific cancer prevalence declines due to preventive advances; hence, as populations age over the coming decades, cancer will become commoner, but mainly among adults older than 65 years who will tend to have more age-related frailties than those diagnosed (younger) in the past (18). On the positive side, this greater longevity partly reflects improved disease prevention and wellness (19) (“healthy aging”, “delayed aging”), just as falling fertility may arise to some extent from more effective contraception (20).

Whatever the reasons for population aging, a key consequence for today’s oncologists is that their future (older) patients – who despite healthy aging are likely to have on average more restricted activities of daily living, more tenuous quality of life, and more competing causes of death (18) – may come to value autonomy and life quality relatively more highly than did their survival-focused predecessors (21, 22). The advent of patient-reported outcome measures represents a major step forward in this process of change, signaling as it does a ‘personalisation’ not of treatment targeting but of quality-of-life feedback and optimisation. Although such changes will not transform practice in the present decade, by 2040 evidence of this transition is predicted to become clear (23).

A different threat to human livelihoods is environmental degradation (24). There may seem little that links oncology practice and the causes or effects of environmental decline; yet when all correlates of the latter problem are considered – global warming, weather disasters, species displacements driving risks of disease spread to humans, travel restrictions, decarbonisation costs, energy shortages, rising prices, geopolitical instability – a total disconnect seems unlikely (25). At the least, traditional access to funding and philanthropy is likely to be constrained by competing cost-intensive problems like climate change (26), with the result – amplified by population aging – that resources for cancer become squeezed. Hence, although paradigm-shifting therapeutic advances must continue to add benefit for cancer patients in the future, other emerging challenges are likely to diminish investment in cancer therapeutics over time, especially when expressed as a fraction of world expenditures against all threats.

Public acceptance of this new reality could bring with it a slow change from an individualistic view of health as priceless (27) to a more socially cognizant “doughnut economics” (28), with proposals of this kind having already been made (29). Such a trend is also consistent with the Moonshot initiative which, in contrast to the War on Cancer, is not driven by major new funds; instead, the Moonshot proposes a switch of emphasis limited to an additional 5% of existing funding, with prevention as the priority. A transition of this kind aligns with recommendations to invest in a Culture of Health (30), based on a mindset valuing shared community needs (31, 32) at least as highly as the ‘magic bullet’ hope and hype which has for so long energised cancer research (33).

## Steps to a greening of oncology

Different cultures of oncology already exist in different parts of the world, implying that changes are possible in any knowledge system (34). For cancer care to evolve from its golden age values to a more equable and lower-profile green age culture (**Table 1**), resistance may be eased by educational campaigns which can convince both public and professionals that these selection pressures reflect essential adaptive challenges for modern healthcare (35). A stepwise approach to this cultural transition is suggested below.

**Table 1:** Comparison of variables proposed to differ between the past (golden age) and potential future (green age) of oncology.

Variable	Golden age	Green age
Public/professional outlook	Only the best cancer treatment is good enough	Good enough cancer treatment may be the best
Clinical research study design	Largely specified by drug companies	Involves payers and patient representatives
Preferred site of standard care	Comprehensive Cancer Center	Community Cancer Center
Cancer patient management	Led throughout by Medical Oncologist	Shared from the start amongst MDT* members
Clinical decision-making	Based mainly on drug trial survival data	Based on nuanced patient-centric algorithms**
Attention to quality of life	Late, reactive to symptoms, 'damage control'	Early, anticipatory, preventive, maintenance
Patient mental health care	As needed, often Psychiatry-based	Pro-active, initially Psychology-based

*MDT, multidisciplinary team meeting, including at least one Medical Oncologist, plus additional Oncologists (Medical, Radiation, and/or Surgical), and at least one Supportive & Palliative Care specialist, and/or Pain Care specialist, Gerontologist, plus at least one Psychiatrist/Psychologist, Oncology Nurse Practitioner (including Stoma Care, Breast Care, etc.), Dietitian, Social Worker, Physiotherapist/Rehabilitation/Exercise Physiologist. **Including not only drug trial survival data (adjusted for level of evidence, statistical power, interpretability of study design, prospective vs. retrospective, primary endpoints, overall vs. disease-free survival, presence or absence of patient crossover, etc.), short- and long-term drug toxicity and tolerability, and quality of life, as per ASCO Value Framework and ESMO-MCBS (see text for references); plus financial costs, both absolute and out-of-pocket; patient convenience; patient autonomy; and other patient preferences; as predicted with or without a given treatment, and compared with other options, including no treatment.

### 1. Win the battle of ideas over good-enough cancer treatment

Change in oncology is slowed by the idea that drug treatment choice – whether right [i.e., best, usually the latest [36)] or wrong (anything other than the best) – is a main determinant of disease outcome. As shown by the small absolute size of most clinical trial benefits, however, the reality is that cancer biology still tends to be the main factor affecting outcomes, with choices between approved treatments [or differences in national expenditures thereon (37)] usually making only modest differences (38). The modern incrementalist culture may exploit patients’ fear of death (39) by perpetuating the norm that only the best is good enough (40), leading to the paradox that a disproportionate amount of oncology costs are generated in the last months of life (41). Such thinking may reflect a survival-of-the-fittest instinct, whereby any threat triggers a safety-first (kill it before it kills you) reaction; yet in the real world less extreme responses may not only suffice, but also prove less morbid (42).

### 2. Re-prioritise from research-centered to patient-centered practice

Focusing solely on the disease-modifying potential of therapies – with the potentially gratifying but often undetectable benefits of immune checkpoint therapy being a case in point (43) – distracts from the importance of factoring in other decision-making criteria such as treatment tolerability, safety, convenience, cost, options of delaying treatment (e.g., by later sequential or crossover drug use), and so on (44). One way to break this cycle, and thus to assess the validity or otherwise of good-enough treatment, is to quantify all factors pertinent to a cancer patient’s predicted length and quality of life with or without the prescription of a treatment (45). If this exercise becomes possible to score – a technical quantum leap not yet reduced to practice – a good-enough cancer therapy could, paradoxically, deliver superior overall (i.e., holistic) outcomes compared to the best cancer treatment as determined by survival data. One step towards this has been the use of clinical trial or gene expression data to predict when addition of adjuvant cytotoxic therapy to hormone therapy delivers such a low absolute breast cancer survival increment as to be not recommendable as a standard of care (46). Validated algorithms which can quantify a given patients’ preferences and priorities thus seem a prerequisite for progress.

### 3. Abandon zero-sum mindsets

Career success in newer subspecialties, such as oncology, has long been favoured by a tight focus on established (homophilic) goals and colleagues (47) – the so-called silo effect – rather than by more collaborative approaches (48). Yet if it is assumed that the problems of oncology do not overlap with those of other important contextual issues, such as the aging population or environmental deterioration, then a zero-sum interaction is assured; that is, any competition for resources between these fields will proceed on an ‘I-win-you-lose’ basis. In contrast, if common goals can be discerned, win-win scenarios for mutual benefit, and hence for co-resourcing, could be pursued (49, 50). Examples of integrated initiatives for cancer patients and the public include:

- Smoking elimination, clean air, ambient toxin reduction (e.g., radon, asbestos)
- Addiction prevention, mental resilience promotion
- Vaccination drives (e.g., HPV, HBV)
- Exercise programs (51)
- Ideal body weight education
- Food/beverage labeling improvements
- More multidisciplinary hospital-based care
- More community-based care for standard clinical problems
- User-friendly software development to aid more nuanced decision-making

### 4. Question self-reinforcing feedback loops

A risk of any golden age is that it selects for its own survival. In oncology, therapeutic progress has always been similarly sought by physicians, patients, pharmas, philanthropists, and the press; the only stakeholders who are motivated to query such progress are the third-party payers (52). This near-unanimity over the desirability of constant progress has made objective debate difficult (53), with the careers of critics prone to damage (54). The best solution to this problem will be to develop and validate quantitative metrics that can value holistic (i.e., qualitative) patient-centered variables, thus extending and refining the meaning of cost-efficacy (55).

### 5. Broaden decision-making and management

This quest to validate predictors of therapeutic value has made progress with upgradings of both the American Society of Clinical Oncology (ASCO) Value Framework Net Benefit Score (56) and the European Society of Medical Oncology (ESMO) Magnitude of Clinical Benefit Scale (MCBS) (57). The pros and cons of these sophisticated tools have been compared (58), and reveal promising complementarity in the questions addressed (59). Implementation of these algorithms remains labour-intensive, however, hampering patient inputs and clinical adoption (60). Broadening management to include routine early involvement of non-oncology multidisciplinary experts should help to dilute what is now, for many standard-setting cancer patients, an overly specialised approach to care (61). Better patient experiences could also result from moving standard care away from specialised cancer centers into general hospitals or local communities, assisted where needed by telehealth communications, while also evolving towards value-based reimbursement systems (62). More promotion of oncologists for excellence in communication or education (63) could be another step towards a flatter service structure. Finally, more attention to the mental resilience of patients, will also add value to patients’ well-being, in part by reducing reliance on test results as critical arbiters of survivorship (64).

## Conclusion

The choice of a War on Cancer as an encore to the Apollo moon landings signalled the zenith for cancer as an existential human threat, kicking off a golden age for the science and practice of oncology. Times have since changed, however, as population aging and planetary threats have altered the trajectory of public concerns. The field of oncology is likely to feel these pressures to adapt within the next two decades, and the healthcare changes that result over the next fifty years could prove to be just as important and beneficial as the paradigm shifts that preceded these.

A global approach, based on public and professional education (65), will be needed to bring about economics-based system changes that can adapt to the disruptive evolutionary era ahead (66). Crosstalk with all stakeholders – including, though not limited to, patients, physicians, advocacy groups, governments, insurers, and pharmas – will necessarily precede such changes. The 20^th^ century paradigm of ever more resources (ultimately derived from the environment) being harnessed to deliver ever more personalised oncologic increments (ultimately benefiting individuals more than populations) may prove to be less sustainably applicable to the 21^st^ century world, where an imbalance between human demand and ecosystem fragility has become evident. Constructive change will require that concepts such as ‘greater good’, ‘big picture’ and ‘longer term’ come to be pursued more systematically than the self-interested priorities of the past.

## Author contributions

RE wrote the first draft with inputs from FL and YG, then the second draft was further appraised and critiqued by FL and YG. All authors contributed to the article and approved the submitted version.
